# The meningo-orbital band from an endoscopic transorbital approach: an anatomical study

**DOI:** 10.3389/fnana.2025.1578959

**Published:** 2025-05-14

**Authors:** Roberto Manfrellotti, Dario Gagliano, Roberta Costanzo, Alejandra Mosteiro, Marta Codes Méndez, Doriam Perera Valdivia, Nikolay Lasunin, Carlo Giorgio Giussani, Giorgio Giovanni Carrabba, Joaquim Enseñat, Alberto Di Somma, Alberto Prats-Galino

**Affiliations:** ^1^Faculty of Medicine and Health Sciences, University of Barcelona, Barcelona, Spain; ^2^IRCCS Ca 'Granda Foundation Maggiore Policlinico Hospital, Milan, Italy; ^3^Department of Biomedicine, Neurosciences and Advanced Diagnostics, University of Palermo, Palermo, Italy; ^4^Azienda Ospedaliera Universitaria Policlinico Paolo Giaccone, Palermo, Italy; ^5^Department of Neurosurgery, Clínic Institute of Neurosciences, Barcelona Clinic, Barcelona, Spain; ^6^Military Hospital Dr. Alejandro Dávila Bolaños School, Managua, Nicaragua; ^7^N.N. Burdenko National Scientific and Practical Center for Neurosurgery, Moscow, Russia; ^8^IRCCS San Gerardo dei Tintori Foundation, Monza, Italy

**Keywords:** endoscopic transorbital approach, lesser sphenoid wing, meningo-orbital band, neuroanatomy, skull base

## Abstract

**Introduction:**

The meningo-orbital band (MOB) is an intricate dural structure extending between the periorbita, the frontal dura, and the temporal dura. The endoscopic transorbital approach (ETOA) provides a more thorough understanding of its anatomy.

**Materials and methods:**

Anatomical dissections were performed on 15 human head specimens (30 orbits) at the Laboratory of Surgical Neuroanatomy (LSNA) at the University of Barcelona. The specimens were preserved using a Cambridge solution for optimal fixation. An endoscopic transorbital approach (ETOA) was used to isolate the meningo-orbital band (MOB). A rigid 4-mm endoscope with an HD camera and light source was used for the procedure. Multislice helical CT scans were performed both before and after the dissections to document the anatomical features. Additionally, a specialized software (The ImagingSource®) was used to calculate the variability in the angle between the first two bone pillars of the ETOA: the sagittal crest (SC) and the lesser sphenoid wing (LSW). The vascularization of the MOB was studied by longitudinally cutting the band and using red and blue latex injections into the carotid arteries and jugular veins, respectively, to highlight the cerebral vasculature.

**Results:**

In the endoscopic transorbital approach (ETOA), key structures, including the greater and lesser sphenoid wings, are excised, exposing the meningo-orbital band (MOB). The MOB extends from the periorbita medially to the frontal and temporal dura laterally and is firmly attached to the anterior clinoid process (ACP). Anatomical dissection reveals the MOB’s complex three-dimensional structure and its relationships with cranial nerves III, IV, and V1 along the lateral wall of the cavernous sinus and the superior orbital fissure (SOF). The ACP serves as a protective barrier between the MOB and the paraclinoid segment of the internal carotid artery (ICA). Additionally, the MOB is vascularized by the MOB artery (MOBA), a branch of the middle meningeal artery, which bifurcates into the frontal and temporal branches.

**Conclusion:**

This study highlights the key anatomical relationships of the meningo-orbital band (MOB) with critical structures, including cranial nerves III, IV, and V1, as well as the ICA. These findings are essential for refining surgical planning and improving the safety and precision of skull base surgery.

## Introduction

The meningo-orbital band (MOB), also known as the frontotemporal dural fold (FTDF), is a dural fold tethering the temporal and frontal dura with the periorbita and running along the superolateral portion of the superior orbital fissure (SOF) (Anania et al., 2022; Fukuda et al., 2014; Yoon et al., 2012). The intracranial dura comprises two layers: the periosteal dura and the dura propria. The periosteal dura is attached to the bone and exits through foramina and fissures, continuing to the extracranial periosteum (Fukuda et al., 2014). The MOB can be defined as a periosteal layer situated between the superior and inferior borders of the horizontal limb of the SOF (Saenz et al., 2020).

It was comprehensively and accurately described from a transcranial perspective (Campero et al., 2014); however, a detailed description of the MOB from an endoscopic transorbital approach (ETOA) is still lacking. Only one study examined the MOB from a transorbital perspective since the initial definition of transorbital neuroendoscopy surgery (TONES) in 2007 (Dallan et al., 2017). Nevertheless, the rapid increase in endoscopic transorbital surgeries has required a more comprehensive anatomical description of the MOB from a transorbital perspective.

Furthermore, the MOB could be considered the “anatomical key” that opens the interdural space between the lateral wall of the cavernous sinus (LWCS) and the medial side of the temporal pole. Opening the MOB is a necessary step to begin peeling the cavernous sinus and, therefore, the interdural dissection (Di Somma et al., 2018).

The goal of this anatomy study is to use the ETOA to explore the anatomy of the MOB, focusing on its spatial orientation and relationships with the lesser sphenoid wing (LSW) and the sagittal crest (SC). Additionally, the study will examine the MOB’s neurovascular relationships with the internal carotid artery (ICA), the LWCS, and the oculomotor (III), trochlear (IV), and ophthalmic (V_1_) cranial nerves. Finally, the intrinsic vascularization of the MOB, dependent on the middle meningeal artery (MMA), will also be assessed.

## Materials and methods

### Anatomic dissections

This study was conducted in accordance with international ethical guidelines, including the Declaration of Helsinki (2013 version) and applicable local regulations. Ethical approval was obtained from the Ethics Committee of the University of Barcelona (IRB) and the Clinical Research Ethics Committee (CEIm) of the Hospital Clínic i Provincial de Barcelona, under the registration number Reg. HCB/2024/0919. This Committee complies with the EMA/CHMP/ICH/135/1995 regulations, both in its composition and in its Standard Operating Procedures (SOPs). The specimens used were obtained from voluntary donors who signed informed consent with the University of Barcelona authorizing the use of the samples for anatomical and surgical research (Barcelona, 28 November 2024). Fifteen human head specimens (30 orbits) used in this study consisted of 6 female and 9 male donors, with an average age of 88 and 83 years, respectively. None of the donors had a history of cerebrovascular diseases, either recent or remote, including ischemic or hemorrhagic stroke, acute or chronic subdural hematoma, subarachnoid hemorrhage, or any traumatic brain injury or neuro-oncological pathologies.

Anatomic dissections were performed at the Laboratory of Surgical NeuroAnatomy (LSNA) of the University of Barcelona (Barcelona, Spain). Each specimen was cleaned of any clots accumulated in the cerebral circulation by injecting a saline solution into the common carotid arteries in the neck. Subsequently, keeping the carotid arteries cannulated, the Cambridge solution was injected. This solution was developed in 1985 in Cambridge by Bari Logan, who reduced the concentration of formalin from 10 to 3% and replaced it with methanol. This technique of soft embalming provides greater flexibility and better fixation of the specimen, while also producing fewer harmful fumes (Ohmichi et al., 2006; Blessing et al., 2018; Srivastava and Nagwani, 2019). Finally, human head specimens were injected with red and blue latex into the common carotid artery and jugular vein, respectively, to highlight the arteriovenous cerebral vascular tree.

Before and after dissections, all specimens underwent a multislice helical computed tomography (CT) scan (SIEMENS Somatom GoTop software version VA30A-SP03) with 0.5 mm-thick axial spiral sections and a 0° gantry angle.

An endoscopic endonasal approach was performed using a rigid endoscope of 4 mm diameter and 18 cm length, with 0° lenses (Karl Storz, Tuttlingen, Germany). The endoscope was connected to a light source through a fiber optic cable (300 W Xenon; Karl Storz) and to an HD camera (Endovision Telecam SL; Karl Storz). Moreover, a cutting and diamond drill (πdrive Motor; Stryker), and a suction device were used.

### Endoscopic transorbital approach

A superior eyelid endoscopic transorbital approach was performed as previously described (Di Somma et al., 2018; Di Somma et al., 2022; Almeida et al., 2018; Dallan et al., 2018; De Rosa et al., 2022; Moe et al., 2010). The head was positioned supine on the operating table in a neutral position, with a slight rotation of 5–10° toward the contralateral side so that the lateral orbital wall was oriented in a plane parallel to the direction of the approach. After positioning, a superior eyelid skin incision was planned at a distance of 6–8 mm from the horizontal palpebral fissure in male and 8–10 mm in female individuals; the orbicularis oculi muscle was carefully cut at the precise point where its pre-tarsal portion meets the pre-septal one. Therefore, the incision was extended laterally toward the lateral orbital rim, at the level of the frontozygomatic suture, avoiding crossing the white plane, which consists of the superior tarsus, the orbital septum, and the tendon of the levator palpebrae muscle (levator aponeurosis). An adaptable retractor was inserted to gently separate the contents of the orbit medially, creating an appropriate triangular-shaped operative region, defined by the lateral orbital rim as the side boundary, the retractor as the inner boundary, and the lateral aspect of the upper eyelid crease as the superior boundary. Continuing with the dissection revealed the periorbita, which exposes the orbital surface of the greater sphenoid wing. Drilling the orbital surfaces of the zygomatic bone and the great sphenoid wing revealed the temporal fascia on the outer side of the surgical site. This created a “V”-shaped area bounded by the inferior orbital fissure at the inferior apex, the lesser sphenoid wing at the top, the sagittal crest on the inside, and the sphenoid bone on the outside. As drilling proceeded inside the “V”-shaped area, the temporal dura was gradually exposed. This maneuver allowed for the skeletonization of the LSW, interposing the temporal dura inferiorly and the fontal dura superiorly, and exposing the MOB behind the LSW (Di Somma et al., 2022; Moe et al., 2010; Lopez et al., 2021; Jeon et al., 2019; Almeida et al., 2017).

### Dissection of the MOB

At the end of the skin phase of the ETOA, and once exposure of the temporal and frontal dura was achieved, the MOB was properly isolated and separated from the temporal and frontal dura, inferiorly and superiorly, respectively, as well as from the periorbita, medially, to study its anatomy and neurovascular relationships. Afterward, it was longitudinally cut in a medial-to-lateral direction to unmask its contents and describe its vascularization. Moreover, specialized software (The ImagingSource®) was used to calculate the variability in the angle between the first two bone pillars of the ETOA, namely the sagittal crest (SC) and the lesser sphenoid wing (LSW).

## Results

This anatomical investigation provided a comprehensive description of the anatomy, the neurovascular relationships, and the vascularization of the MOB from an endoscopic transorbital approach.

### Anatomy

The skin phase of the transorbital endoscopic approach was completed as described in the literature, allowing for the highlighting of the temporal dura and frontal dura, the SOF, and the first two bone pillars of the ETOA, namely the sagittal crest (SC) and the lesser wing of the sphenoid (Di Somma et al., 2022).

The SC is a bone ridge triangle that shapes dorsally around the superior orbital fissure, resulting in the residual bone fragment after drilling the lateral aspect of the greater sphenoid wing (GSW). Anatomically, it can be defined by three key landmarks: the base, located at the exit of the maxillary nerve from the foramen rotundum (FR); the anterior edge, an imaginary line passing where the GSW turns from a coronal plane to a sagittal plane; and the posterior free edge, pointing toward the interdural plane of the cavernous sinus (Corrivetti et al., 2022).

Once the LSW is skeletonized and completely removed, the MOB, located between the temporal and frontal dura and immediately behind the LSW, can be visualized. Continuing the dissection reveals the extension of the MOB into the periorbita, its passage through the super-external portion of the superior orbital fissure (SOF), and its juxtaposition at its horizontal segment.

**Figure 1:**
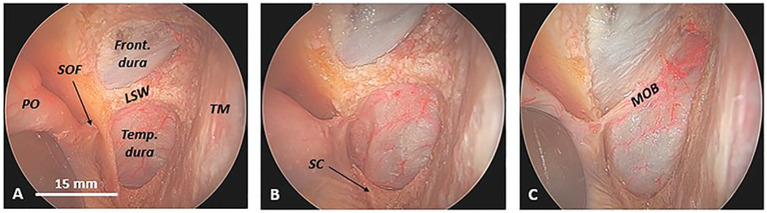
Exposure of the meningo-orbital band in a cadaveric specimen (left side) from an endoscopic transorbital approach. **(A)** Once the temporal dura and frontal dura, the superior orbital fissure, and the sagittal crest (SC) are exposed, the lesser sphenoid wing appears evident between the temporal dura mater, inferiorly, the frontal dura mater, superiorly, and the superior orbital fissure, medially. **(B)** The progressive removal of the anterior wall of the superior orbital fissure and, thus, the sagittal crest exposes the point where the meningo-orbital band inserts into the periorbita and thus the anterior part of the interdural space between the lateral wall of the cavernous sinus, medially, and the medial wall of the temporal dura, laterally. **(C)** The complete drilling of the lesser sphenoid wing is necessary to finally expose the meningo-orbital band, stretched between the temporal and frontal dura and the periorbita. Note the constant relationship between the meningo-orbital band and the first two bone pillars of the transorbital endoscopic approach, namely the sagittal crest and the lesser sphenoid wing. Front. dura, frontal dura; LSW, lesser sphenoid wing; MOB, meningo-orbital band; PO, periorbita; SOF, superior orbital fissure; Temp. dura, temporal dura; TM, temporal muscle.

Once the MOB has been isolated, its constant anatomical relationships with the SC and the LSW become evident (Figure 1).

**Figure 2:**
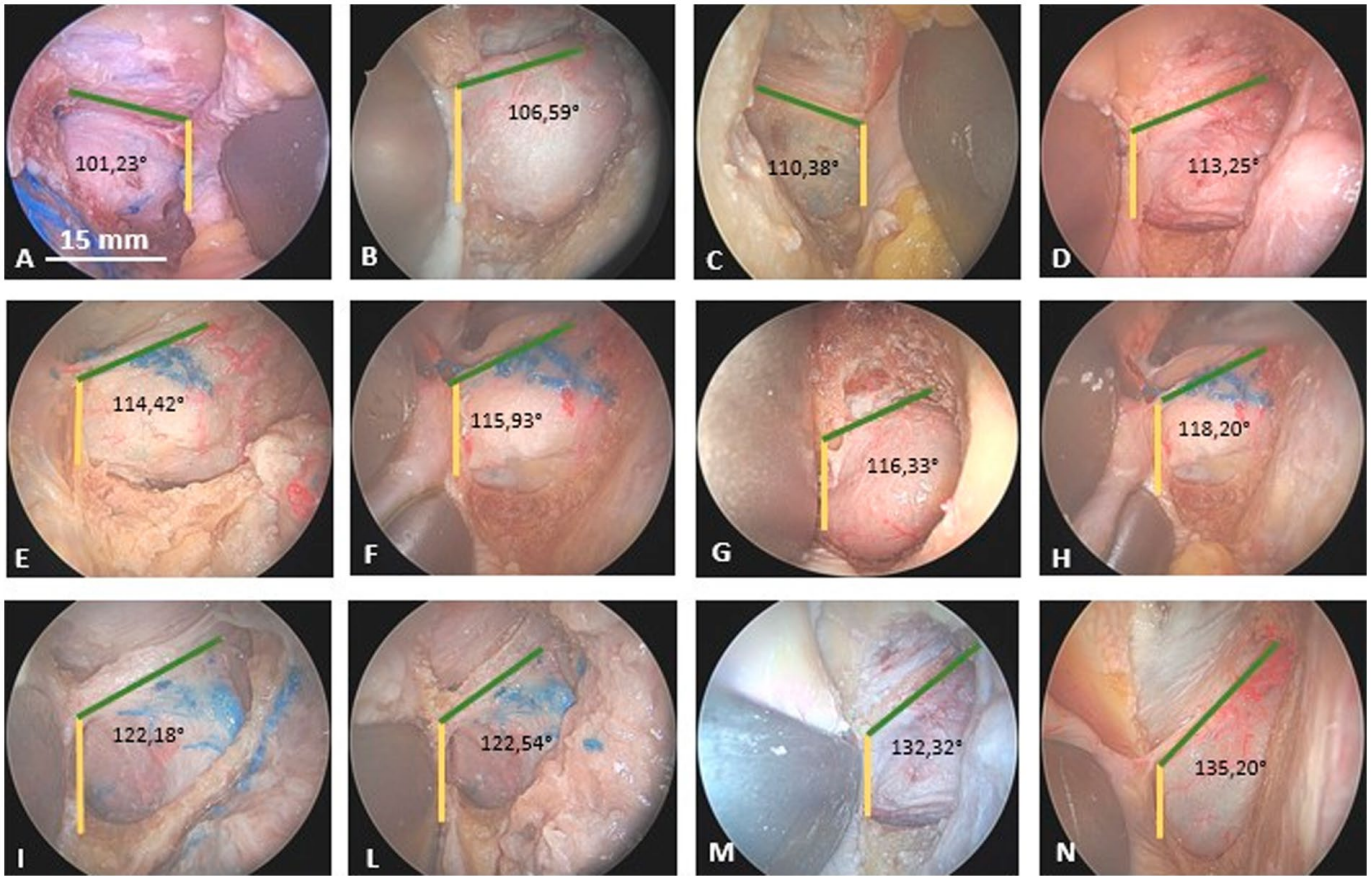
Degree of variability in the angle between the sagittal crest (yellow line) and the meningo-orbital band (green line) in many cadaveric specimens from an endoscopic transorbital perspective. **(A and C)**: right side; **(B,D–L)**: left side. The different specimens are inserted into the panel in relation to the progressively increasing angle between the sagittal crest and the meningo-orbital band (from 101,23° to 135,20°).

However, after opening the SOF once and removing the superior segment of the SC, a significant degree of variability in the angle between the SC and the MOB can be observed. In our investigation, this angle (calculated using the software ImagingSource®) ranges from 101° to 135°, closely aligned with the spatial orientation of the LSW. Therefore, by meticulously observing the junction between the sphenoidal margin of the frontal bone and the LSW, the spatial orientation of the MOB in the coronal plane can be anticipated (Figure 2).

### Neurovascular relationships

The anatomical relationships of the MOB with the optic nerve, the clinoid segment of the internal carotid artery (or segment C5 according to the Bouthillier classification of the internal carotid artery (Bouthillier et al., 1996)), and cranial nerves III, IV, and V_1_ (along the lateral wall of the cavernous sinus) were examined after the MOB was isolated.

**Figure 3:**
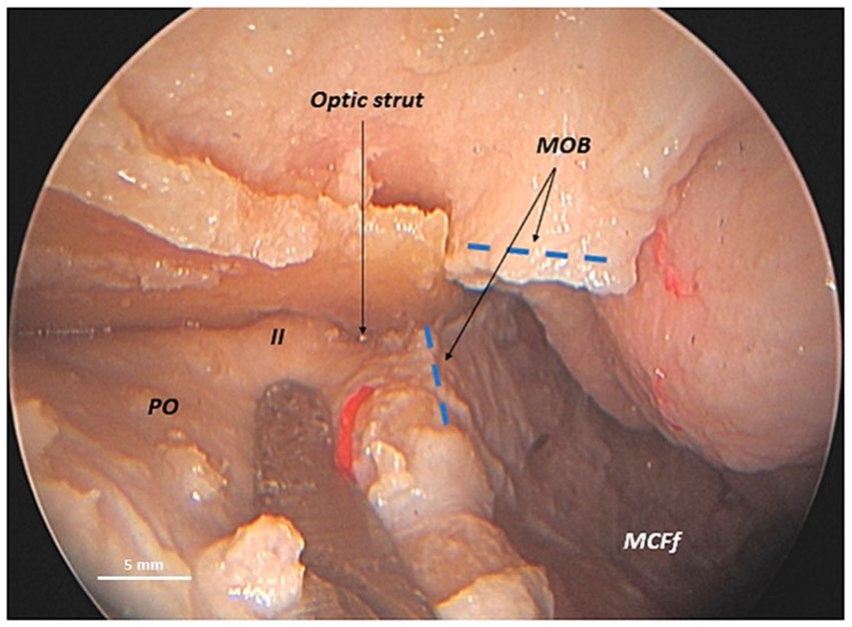
Anatomical relationship between the meningo-orbital band and the optic nerve in a human head specimen (left side) from an endoscopic transorbital perspective. Once the superior orbital fissure is exposed via a transorbital approach and the periorbita is separated from the roof of the orbit, the longitudinal incision of the meningo-orbital band in the anteroposterior direction is completed—from the point of its insertion into the periorbita to its most posterior portion adhering to the posterior tip of the anterior clinoid process. This maneuver enables the interdural dissection between the lateral wall of the cavernous sinus and the mesial surface of the temporal dura and the completion of the exposure of the middle cranial fossa floor. It also enables us to emphasize that the optic strut, the posterior root of the anterior clinoid process, is situated between the meningo-orbital band and the optic nerve, visible within the optic canal. ACP, anterior clinoid process; LWCS, lateral wall of cavernous sinus; MCFf, middle cranial fossa floor; MOB, meningo-orbital band; PO, periorbita; Temp. dura, temporal dura; II, optic nerve (second cranial nerve); asterisk (*), dissector; dashed blue lines, margins, inferior and superior, of the longitudinal incision of the meningo-orbital band.

By partially incising the MOB longitudinally in an anteroposterior direction at the level of the anterior base of the ACP to separate the temporal dura from the periorbita, it becomes feasible to lateromedially detach the periorbita from the orbital roof. Consequently, the posterior root of the anterior clinoid process (ACP), the optic strut, and the optic nerve within the optic canal are sequentially observed, along with the foramen for the posterior ethmoidal artery, in a lateromedial direction (medial to the optic nerve). The optic strut, which defines the inferolateral wall of the optic canal, anatomically separates the optic nerve from the MOB (Figure 3).

**Figure 4:**
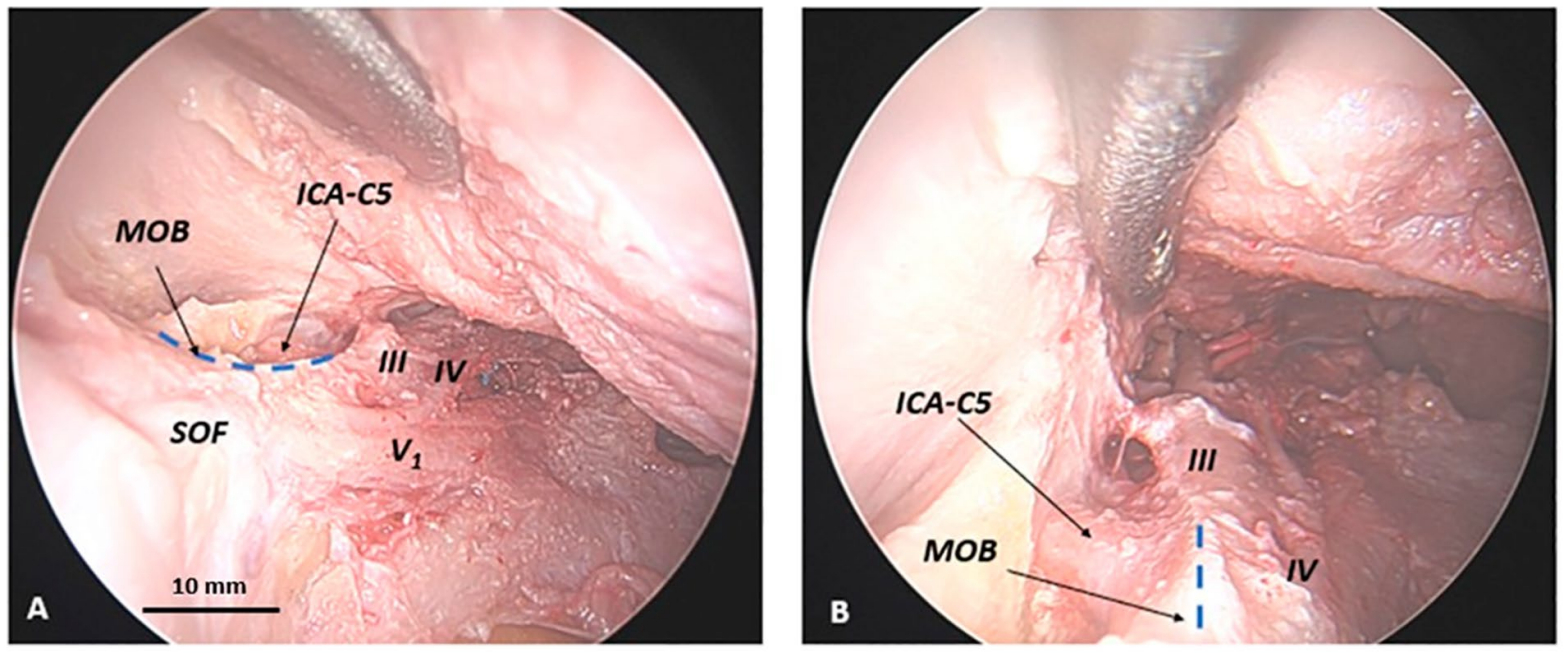
Anatomical relationship between the meningo-orbital band and the clinoidal segment of the internal carotid artery in a human head specimen (left side) from an endoscopic transorbital approach. Upon the completion of the longitudinal incision of the meningo-orbital band, the lateral wall of the cavernous sinus and the middle cranial fossa floor, with the Meckel’s cave and the Gasserian ganglion, must be exposed. The anterior clinoid process must be removed in order to visualize the clinoid segment of the internal carotid artery. In **(A)** lateral view and **(B)** dorsal view, it can be observed how the anterior clinoid process separates the meningo-orbital band and the clinoid carotid (or C5 segment of the internal carotid artery). ICA-C5, clinoid segment (or C5-segment, according to Bouthillier’s classification) of the internal carotid artery; Front. dura, frontal dura; GG, Gasserian ganglion; MOB, meningo-orbital band; SOF, superior orbital fissure; Temp. dura, temporal dura; III, third cranial nerve (oculomotor nerve); IV, fourth cranial nerve (trochlear nerve); V_1_, first branch of the trigeminal nerve (ophthalmic nerve); V_2_, second branch of the trigeminal nerve (maxillary nerve); V_3_, third branch of the trigeminal nerve (mandibular nerve); asterisk (*), dissector; dashed blue line, margin of the longitudinal incision of the meningo-orbital band below the anterior clinoid process (removed in this photo).

To reach the clinoid segment of the internal carotid artery, the MOB must be separated from the ventral surface of the anterior clinoid process. This step is followed by an extradural anterior clinoidectomy, as previously described (Lim et al., 2022). This maneuver confirms the lateral interposition of the ACP between the MOB and the lateral wall of the clinoid segment of the internal carotid artery. Therefore, the ACP acts as a protective barrier, separating the MOB from the carotid artery. The paraclinoid carotid artery is exposed only after the removal of the anterior clinoid process, highlighting the critical role of the ACP in safeguarding the carotid artery during dissection (Figure 4).

### Vascularization

**Figure 5:**
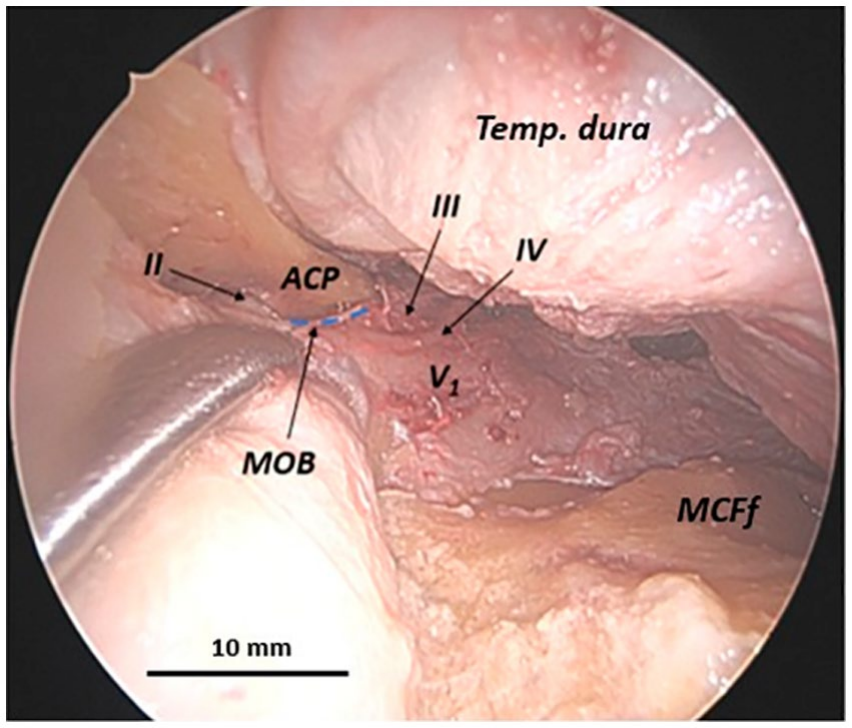
Anatomical relationship between the meningo-orbital band and the oculomotor, trochlear, and ophthalmic nerves along the lateral wall of the cavernous sinus in a human head specimen (left side) from an endoscopic transorbital approach. Once the longitudinal incision of the meningo-orbital band has been completed and the lateral wall of the cavernous sinus has been exposed, the relationships between the deepest portion of the meningo-orbital band, interposed between the periorbita medially, the temporal dura laterally, the ACP superiorly, and the lateral wall of the cavernous sinus posteriorly, become evident. At that level, the meningo-orbital band establishes important relationships with the oculomotor, the trochlear, and the ophthalmic cranial nerves, which run along the wall of the cavernous sinus. ACP, anterior clinoid process; MCFf, middle cranial fossa floor; MOB, meningo-orbital band; Temp. dura, temporal dura; III, third cranial nerve (oculomotor nerve); IV, fourth cranial nerve (trochlear nerve); V_1_, first branch of the trigeminal nerve (ophthalmic nerve); dashed blue line, margin of the longitudinal incision of the meningo-orbital band below the anterior clinoid process.

**Figure 6:**
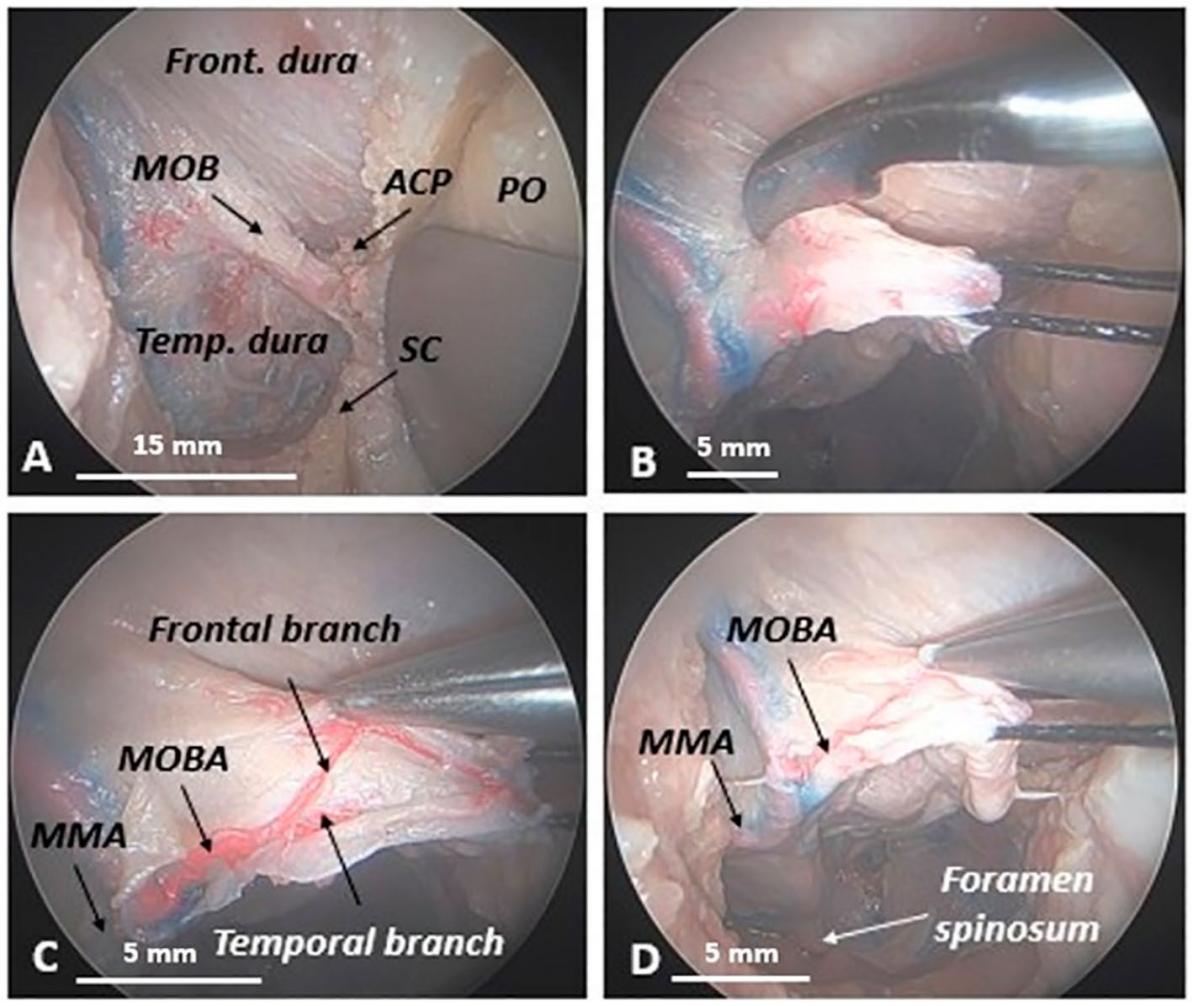
Opening of the meningo-orbital band to highlight its vascularization in a human head specimen (right side) from an endoscopic transorbital approach. **(A)** Upon the removal of the lesser sphenoid wing, the meningo-orbital band appears as a dural fold, interconnecting the frontal and temporal dura with the periorbita. **(B)** Transverse incision of the MOB in a latero-medial direction (in this photo, a suture thread used to retract the MOB, once detached from the temporal dura and frontal dura, is also visible). **(C)** The opening of the MOB shows that it contains a twisted artery, which we have named the meningo-orbital band artery or MOBA. This artery splits into two branches, one higher or frontal and one lower or temporal, which correspond to the frontal and temporal parts of the MOB. **(D)** Anatomical evidence demonstrates that the MOBA originates from the middle meningeal artery, passing through the spinous foramen.

We used a microsurgical technique to longitudinally incise the MOB in a mediolateral direction, investigating its vascularization. This maneuver elegantly unveils the MOB, resembling the opening of a book, and allows for a detailed exploration of its contents. Opening the MOB revealed the presence of an arterial structure within, which we have identified and termed the MOB artery (MOBA), a significant finding that enhances our understanding of its anatomy. During the mediolateral dissection of the MOBA, we uncovered its origin at the level of the middle meningeal artery (MMA) (Figures 5, 6).

**Figure 7:**
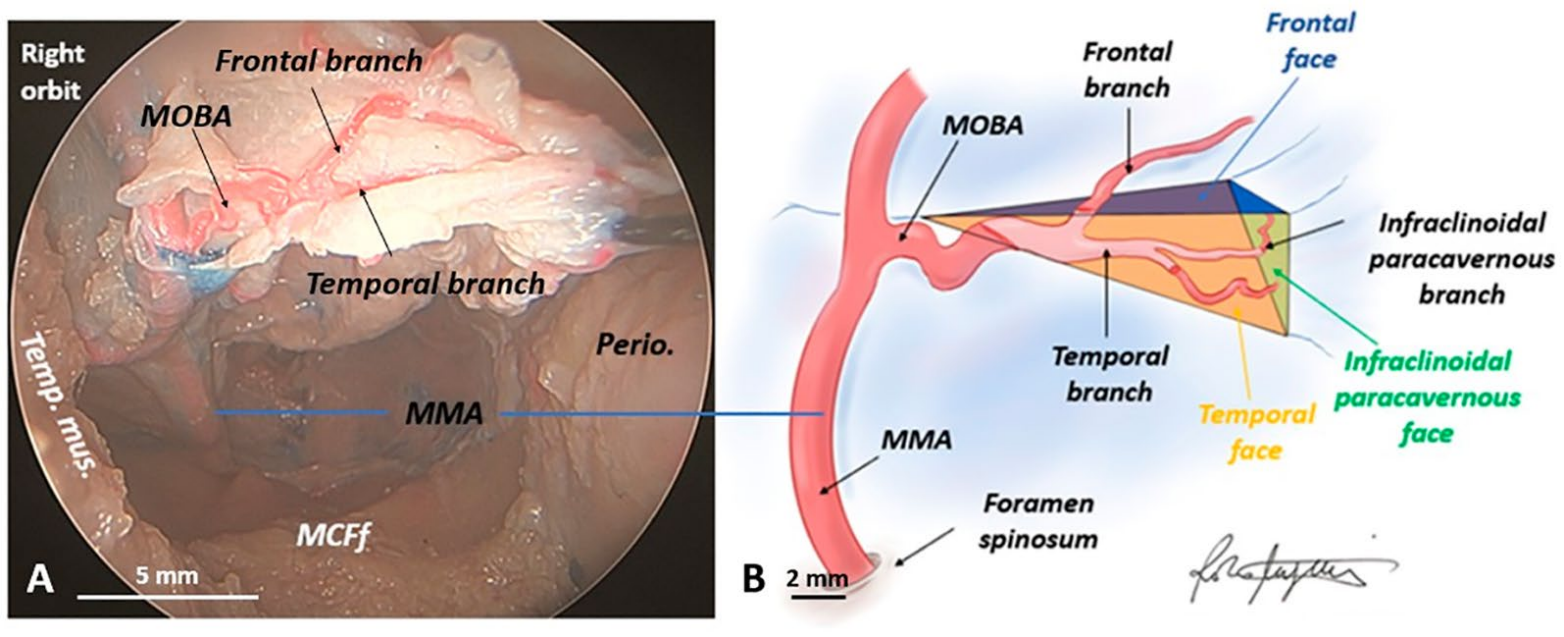
Vascularization of the meningo-orbital band in a human head specimen (right side) from an endoscopic transorbital approach. **(A)** Microsurgical dissection of the meningo-orbital band and demonstration of how the meningo-orbital band artery, originating from the middle meningeal artery, bifurcates within the meningo-orbital band into a superior or frontal branch and an inferior or temporal branch. **(B)** Schematic drawing depicting the vascularization of the meningo-orbital band (performed by R.M.). In detail, the meningo-orbital band artery, originating from the middle meningeal artery, in 100% of specimens bifurcates into an upper or frontal branch and a lower or temporal one; moreover, the lower branch undergoes an additional bifurcation, which gives rise, in 40% of specimens, to an infraclinoidal paracavernous branch, which runs along the lateral wall of the cavernous sinus, inferior to the anterior clinoid process. MCFf, middle cranial fossa floor; MMA, middle meningeal artery; MOBA, meningo-orbital band artery; Perio., periorbita; Temp. mus., temporal muscle.

Continuing to trace the MOBA along its entire length within the MOB in a lateral-to-medial direction elegantly revealed its tortuous course and subsequent bifurcation into two branches—superior and inferior—which we have designated as frontal and temporal branches, respectively. All specimens examined exhibited this distinct vascular pattern within the MOB. However, in 40% of cases, we observed an additional bifurcation of the lower (temporal) branch into two smaller branches. One of these branches was designated the infraclinoidal paracavernous branch, due to its course along the lateral wall of the cavernous sinus and the ventral surface of the anterior clinoid process (Figure 7).

Furthermore, our in-depth understanding of the MOB’s three-dimensional structure has been significantly enhanced by a detailed exploration of its vascularization. The branches of the MOBA within the MOB serve as confirmation of the complexity of its three-dimensional anatomy. Nestled between the periorbita medially and the temporal and frontal dura laterally, the MOB takes on the form of a pyramid with a triangular medial base, transcending the simplicity of a mere dural fold. This pyramidal structure is defined by its triangular base: the superior or frontal face, the anterior or sphenoidal face (so named due to its proximity to the LSW, as previously discussed), the inferior or temporal face, and the lateral apex, where the MOBA originates from the MMA. Three distinct margins can be delineated within the MOB: two anterior—superior (frontal) and inferior (temporal)—and one posterior (temporo-frontal). Remarkably, the vascularization of the MOB corresponds precisely to its complex three-dimensional form. The superior branch of the MOBA, also known as the frontal branch, ascends and traces the superior face of the MOB before reaching the frontal dura. The inferior or temporal branch runs along the anterior face of the MOB, dorsal to the LSW. Notably, in 40% of cases, this inferior branch gives rise to a secondary vessel, the paracavernous infraclinoid, which runs parallel to the lateral wall of the cavernous sinus and lies inferior to the ACP, thus defining the triangular, medial base of the MOB. The base itself, triangular in shape, features three key vertices: the superior (frontal) and inferior (temporal) anterior vertices and the posterior vertex, which reaches the tip of the ACP, where the oculomotor nerve contacts the ventral surface of the ACP along the lateral wall of the cavernous sinus.

## Discussion

The shift from Dolenc’s classical extradural anterior clinoidectomy, which did not incorporate the incision of the meningo-orbital band (MOB), to the modern trans-SOF extradural approach represents a fundamental leap in skull base surgery. This progression is intrinsically tied to a deeper understanding of the MOB’s anatomy and its critical role in facilitating safer and more effective surgical strategies (Coscarella et al., 2003; Ammirati and Bernardo, 2007; Noguchi et al., 2005; Kinzel et al., 2015).

Dolenc’s original technique, introduced in 1985, used an extensive transcavernous approach to access the paraclinoid region without detaching the MOB or peeling the LWCS (Dolenc, 1985; Dolenc, 2003). Dolenc’s approach, while groundbreaking at the time, was limited by the inability to fully expose the ACP and adjacent structures, which required blind dissection and increased the risk of injury to surrounding neurovascular elements. The key limitation was the obstruction of deeper areas by the intact MOB, which hindered access to critical areas such as the lateral wall of the cavernous sinus.

In our study, we present how a refined understanding of the MOB’s anatomy—especially its attachment to the lateral edge of the SOF and its close relationship with the periorbita and the dura of the lateral cavernous sinus wall—has catalyzed the evolution from the traditional approach to the more modern extradural trans-SOF approach (Coscarella et al., 2003; Mori, 2022).

By incising the MOB, the surgeon gains direct and unobstructed access to the lateral cavernous sinus, significantly improving the ability to safely peel the sinus wall.

The role of the MOB in enhancing surgical exposure is not limited to facilitating safe peeling of the cavernous sinus; its detailed anatomical knowledge also enables surgeons to better navigate the challenging frontotemporal dura and periorbita. Opening the MOB creates a clear corridor to the lateral cavernous sinus, improving surgical orientation and allowing for a more precise and safer dissection. This approach not only enhances visualization but also minimizes the risk of thermal injury or mechanical damage to the surrounding tissues by reducing reliance on high-powered drills in confined spaces (Coscarella et al., 2003; Froelich et al., 2007; Francois et al., 2010).

Our findings confirm that opening the meningo-orbital band (MOB) is a crucial step in the trans-superior orbital fissure (trans-SOF) approach. This maneuver, guided by precise anatomical landmarks, has allowed a shift from earlier blind dissections to more refined and safer techniques, significantly improving the outcomes of anterior clinoidectomy by enabling the controlled removal of the anterior clinoid process and the unroofing of the optic canal (Janjua et al., 2008).

In the extradural trans-SOF approach, the MOB is incised for 3–5 mm along the temporal meningeal dura to avoid periorbital injury (Harris and Rhoton, 1976). Dissection typically proceeds from the anterior border of the greater sphenoid wing (GSW) toward the lesser sphenoid wing (LSW) and the base of the anterior clinoid process (ACP) (Noguchi et al., 2005; Gonçalves et al., 2012). Anatomical studies, including Anania et al. (2022), have shown that this dissection can be conducted safely without damaging the cranial nerve or the cavernous sinus, provided the incision remains limited in depth.

Coscarella et al. (2003) recommended initiating the MOB incision toward the foramen rotundum to facilitate dura elevation and protect SOF contents. Fukuda and Saenz proposed stepwise approaches to MOB dissection, ranging from complete detachment and lateral wall removal (Fukuda et al., 2014) to simplified sequences involving shallow MOB incision and ACP exposure (Saenz et al., 2020).

Overall, our study supports the growing evidence that accurate MOB handling is key to safer skull base access. The transition from Dolenc’s original technique to the current trans-SOF method exemplifies how anatomical knowledge drives surgical evolution. In particular, the endoscopic transorbital perspective—consistent with Lim et al. (2022)—offers enhanced control and visualization, improving surgical precision and reducing complications.

Moreover, our endoscopic transorbital investigation reveals the MOB as a complex, pyramidal dural structure located between the temporal and frontal dura laterally and the periorbita medially. Its incision grants access to the interdural space, improving visualization of the ACP and the SOF. We found that the angle between the sagittal crest (SC) and MOB ranges from 101° to 135°, closely matching the orientation of the lesser sphenoid wing (LSW), which can serve as a reliable landmark during surgery.

### Relationships between the MOB and cranial nerves along the LWCS

The intracranial dura consists of two layers that could be separated along the lateral wall of the cavernous sinus: the outer layer, the periosteal dura, which covers the inner surface of the osseous skull, and the inner layer, the meningeal dura or dura propria, lined along the brain (Fukuda et al., 2014; Janjua et al., 2008).

The LWCS is formed by the periosteal dural layer containing the III, IV, and V_1_ cranial nerves on their way to the SOF (Saenz et al., 2020), and it was found to be formed by the sleeves or sheaths of dura mater accompanying the corresponding nerves from their points of penetration into the sinus walls (Harris and Rhoton, 1976). Once the SOF is passed, the periosteal dural layer continues into the periorbita and then into the covering of the pericranium.

The MOB is defined as a fused periosteal dural layer between the inferior border and the superior border of the lateral portion of the SOF (Fukuda et al., 2014) and it continues into the lateral wall of the cavernous sinus, where it establishes intimate anatomical relations with the epineurium of cranial nerves III, IV, and V_1_. The opening of the MOB allows for the exposure of the interdural space situated between the periosteal dura externally and the meningeal dura internally. This space, at the level of the lateral wall of the cavernous sinus, contains an intermediate dural layer, called the fibrous layer, which allows the separation of the periosteal dura from the meningeal dura (Gonçalves et al., 2012). This maneuver highlights the relationships between the deepest portion of the MOB, interposed between the periorbita medially, the temporal dura laterally, the ACP superiorly, and the lateral wall of the cavernous sinus posteriorly. In its deepest part, the MOB establishes important relationships with the lateral wall of the cavernous sinus and, therefore, with the cranial nerves oculomotor (III), trochlear (IV), and ophthalmic (V_1_), which run along the wall of the cavernous sinus (Campero et al., 2010).

A detailed understanding of the anatomical interplay between the meningo-orbital band (MOB) and cranial nerves III, IV, and V_1_ is crucial to avoid iatrogenic injury during surgical access to the lateral cavernous sinus. In the endoscopic transorbital approach, the dissection begins inferomedially with a longitudinal incision along the ventral surface of the anterior clinoid process (ACP), progressively exposing the interdural space. While cranial nerves IV and V_1_ are visualized early, the oculomotor nerve (III) remains concealed beneath the deepest portion of the MOB, which is tightly adherent to the ventral surface of the ACP. This intimate anatomical relationship makes nerve III particularly vulnerable. Only after careful and controlled peeling of this dural layer can the nerve be safely exposed, highlighting the importance of meticulous technique in navigating this critical region.

### MOBA and the recurrent meningeal branch of the lacrimal artery

Our study has identified a unique vascular structure, termed meningo-orbital band artery (MOBA), which originates from the MMA. The MMA runs along the inferior surface of the greater sphenoid wing into the MOB, traverses the superior orbital fissure (SOF), and provides vascular supply to the dura of the anterior temporal fossa and the SOF (Bonasia et al., 2020). MMA could send two branches into orbit (Martins et al., 2005; Akdemir Aktas et al., 2020). One branch enters the orbit through the superior orbital fissure (SOF) and anastomoses with the recurrent meningeal branch of the lacrimal artery, a branch of the ophthalmic artery. This anastomosis gives rise to the recurrent meningeal branch of the lacrimal artery (LA), which represents a critical connection between the external and internal carotid systems (Erdogmus and Govsa, 2005). A second orbital branch of the MMA can pass through the cranio-orbital foramen (also known as the foramen of Hyrtl) as the meningolacrimal artery, which also establishes an anastomosis with the lacrimal artery. These connections reflect remnants of embryological vascular channels and form part of the potential collateral circulation between the MMA (external carotid system) and the ophthalmic artery (internal carotid system) (Perrini et al., 2007; Lasjaunias et al., 2001). The recurrent meningeal branch of LA originates from the lacrimal artery (LA), a branch of OA, in 85.1% of cases, 8.5% from the anastomotic branch with MMA, and 6.4% directly from the OA (Akdemir Aktas et al., 2020). The recurrent meningeal branch is typically thin and tortuous, contributing minimally to the orbital blood supply, although, in certain cases, it may represent the primary source (Hayreh, 2006).

While the MOBA shares similarities with the recurrent meningeal branch of the LA, particularly in its MMA origin and passage through the SOF, its unique course within the MOB and specific branching pattern distinguish it as a potentially separate anatomical entity. Specifically, the MOBA’s course through the MOB and its unique branching within this dural structure have not been described for the recurrent meningeal branch of LA. Given that angiography is suboptimal for assessing such anastomoses, further studies combining anatomical dissections with advanced imaging techniques will be crucial to definitively classify the MOBA as either a variant of the recurrent meningeal branch of the LA or a distinct anatomical structure.

Understanding the MOBA’s anatomy and its potential role in vascular supply to the orbit and cavernous sinus is essential for safe and successful endovascular and surgical procedures in this region. The MOBA’s distinct anatomical features, coupled with its proximity to critical neurovascular structures such as cranial nerves and the lateral wall of the cavernous sinus, make it an important consideration in transorbital endoscopic approaches to the skull base. Further investigation into its precise role and the risk it may pose during surgical interventions is warranted to refine surgical strategies and minimize complications (Perrini et al., 2007; Bertelli et al., 2017). Ultimately, only targeted neuroanatomical and angiographic studies of the recurrent meningeal branch of LA (Gailloud et al., 2009; Bertelli et al., 2016) will be able to clarify whether the MOBA represents a true anatomical variant or a previously undescribed independent arterial entity.

## Conclusion

This study provides a comprehensive analysis of the meningo-orbital band (MOB), highlighting its three-dimensional configuration and detailing its anatomical relationships with key adjacent structures, including the superior orbital fissure (SOF), the lateral wall of the cavernous sinus (LWCS), the carotid segment of the internal carotid artery, and cranial nerves II (optic nerve), III (oculomotor nerve), IV (trochlear nerve), and V_1_ (ophthalmic branch of the trigeminal nerve).

Furthermore, our findings emphasize that, during the dissection phase of the MOB, it is crucial not only to consider the artery running within the MOB (which we have named the MOBA) and its branching pattern but also to account for the anatomical relationships of the MOB with the nerves of the lateral wall of the cavernous sinus. Of particular importance is the close relationship between the MOB and the oculomotor nerve, which is intimately associated with the ventral portion of the anterior clinoid process, where the MOB attaches in its most distal part.

Additionally, it is important to note that during the dissection of the MOB, the anterior clinoid process serves to protect the clinoid segment of the internal carotid artery.

These findings contribute significantly to both theoretical knowledge and practical applications in skull base surgery, particularly by refining surgical planning and minimizing intraoperative risks. The targeted incision of the MOB is a critical step in the transorbital endoscopic approach, enabling safer and more precise access to the lateral wall of the cavernous sinus and the middle cranial fossa.

Integrating this technique into clinical practice has the potential to advance the management of challenging skull-base lesions. Our study confirms that a thorough understanding and careful handling of the MOB are fundamental to the success of minimally invasive skull base surgery.

## Study limitations

As with other cadaveric anatomic studies, it must be emphasized that differences in tissue characteristics, bleeding, and tolerance to retraction should be considered before extrapolating the results to surgical practice.

## Data Availability

The original contributions presented in the study are included in the article/supplementary material, further inquiries can be directed to the corresponding author.
